# Whole-exome sequencing in obstructive coronary artery disease identifies rare and novel variants in cardiac arrhythmia and pulmonary arterial hypertension–associated genes

**DOI:** 10.17305/bb.2026.13200

**Published:** 2026-01-21

**Authors:** Mohammad Fahad Ullah, Rashid Mir, Jamsheed Javid, Imadeldin Elfaki, Faisal H Altemani, Jameel Barnawi, Naseh A Algehainy, Mohammed M Jalal, Malik A Altayar, Salem Owaid Albalawi, Syed Khalid Mustafa, Aadil Yousif, Eram Husain, Faris J Tayeb, Faisel M AbuDuhier

**Affiliations:** 1 Department of Medical Laboratory Technology, Faculty of Applied Medical Sciences, University of Tabuk, Tabuk, Saudi Arabia; 2 Prince Fahad Bin Sultan Chair for Biomedical Research, Faculty of Applied Medical Sciences, University of Tabuk, Tabuk, Saudi Arabia; 3 Department of Biochemistry, Faculty of Science, University of Tabuk, Tabuk, Saudi Arabia; 4 Department of Cardiology, King Fahd Specialist Hospital, Tabuk, Saudi Arabia; 5 Department of Chemistry, Faculty of Science, University of Tabuk, Tabuk, Saudi Arabia

**Keywords:** Whole exome sequencing, coronary artery disease, familial hypercholesterolemia, cardiac arrhythmias, pulmonary arterial hypertension, genetic variations, human phenotype

## Abstract

Coronary artery disease (CAD) represents a complex interplay of genetic, environmental, and lifestyle factors. In this study, we utilized whole-exome sequencing (WES) on 28 patients with obstructive CAD to identify rare variants that may influence clinical outcomes beyond conventional atherosclerotic risk. We examined 74 genes curated from the Genomics England PanelApp, focusing on familial hypercholesterolemia (FH), cardiac arrhythmias (CA), and pulmonary arterial hypertension (PAH), ultimately detecting 8,251 variants. After applying a stringent filtering process with a population maximum allele frequency (PopMax AF) threshold of <0.1%, we identified 68 candidate variants across 23 genes. The majority were associated with CA (47/68, 69%), followed by PAH (12/68, 18%) and FH (9/68, 13%). Notably, 30 variants (44%) were novel, and 18 were categorized as high-impact frameshift mutations. The highest burden of candidate variants was found in the sodium voltage-gated channel alpha subunit 10 (*SCN10A*), followed by the ryanodine receptor 2 (*RYR2*), mitochondrial seryl-tRNA synthetase 2 (*SARS2*), A-kinase anchoring protein 9 (*AKAP9*), and hyperpolarization-activated cyclic nucleotide-gated channel 4 (*HCN4*). Clinical evaluation revealed a pathogenic variant in the low-density lipoprotein receptor (*LDLR*) and likely pathogenic variants in sodium voltage-gated channel alpha subunit 5 (*SCN5A*) and potassium voltage-gated channel subfamily Q member 1 (*KCNQ1*); additionally, nine other variants were predicted to be deleterious, including five novel *SCN10A* variants. Functional annotation using Gene Ontology (GO) and Human Phenotype Ontology (HPO) highlighted mechanisms impacting cardiac structure, electrical conduction, and lipid homeostasis.

## Introduction

Cardiovascular diseases (CVDs), particularly coronary artery disease (CAD), constitute a leading cause of global morbidity and mortality, accounting for approximately 17.9 million deaths annually [1]. The pathogenesis of CAD is multifactorial, involving a complex interplay of genetic predisposition, environmental factors, and lifestyle choices, including hypertension, hyperlipidemia, obesity, and type 2 diabetes mellitus (T2DM) [2]. Despite advances in diagnostics and therapeutics, a substantial proportion of CAD cases display unexplained genetic susceptibility, indicating the potential role of rare and novel genetic variants in disease progression. Recent innovations in next-generation sequencing (NGS), particularly whole-exome sequencing (WES), have transformed the identification of disease-associated genetic variants, facilitating a deeper understanding of the molecular mechanisms underlying complex disorders like CAD [3]. WES offers a high-resolution approach for detecting coding-region variants, including pathogenic and likely pathogenic mutations that may influence disease risk and progression. Prior studies have implicated genes related to familial hypercholesterolemia (FH), cardiac arrhythmias, and pulmonary arterial hypertension (PAH) in CAD pathogenesis; however, the complete spectrum of genetic contributors remains inadequately characterized [4, 5].

This study aimed to identify rare and novel variants with potential clinical significance and to characterize their functional impact through gene ontology (GO) and human phenotype ontology (HPO) analyses. We employed whole-exome sequencing in a CAD cohort to systematically analyze 74 genes across three clinically relevant panels (FH, cardiac arrhythmias, and PAH)**.** By integrating genomic discovery with phenotypic annotation, this research contributes to the understanding of CAD’s genetic architecture and establishes a framework for translating WES findings into clinical practice.

## Materials and methods

### CAD patient cohort

The research adhered to the Helsinki Declaration guidelines and received ethical approval from the University of Tabuk Research Ethics Committee under reference number (# UT-91-23-2020). Twenty-eight patients with confirmed CAD participated in this study, each of whom provided informed consent. Participants were selected from the King Fahad Specialist Hospital, Tabuk, KSA, and underwent elective angiography for the diagnosis of stable angina. Various clinical assessments were conducted, including X-rays, exercise stress tests, myocardial perfusion imaging, ambulatory electrocardiography, Holter monitoring, chest echocardiograms, computerized tomography coronary angiography, and multigated acquisition scans (MUGA). CAD was defined based on clinical symptoms (stable or unstable angina) and corroborated by invasive coronary angiography, with inclusion criteria requiring macrovascular disease with ≥50% luminal diameter stenosis in at least one of the three major coronary arteries (LAD, LCx, or RCA). These 28 patients were selected consecutively from a larger registry to ensure a representative sample of symptomatic obstructive CAD. Clinical diagnoses of specific arrhythmias, PAH, or FH were not inclusion criteria, and these phenotypes were not systematically assessed.

### Genomic DNA preparation and sequencing

High-quality human genomic DNA was utilized as the source material for this investigation. Library preparation followed the protocol outlined in the Twist Human Core Exome 2.0 Kit manual. Sequencing was performed on the Illumina NovaSeq 6000 platform, adhering to the manufacturer’s recommended procedures.

### Quality control and preprocessing of sequencing data

The initial quality of raw sequencing reads was assessed using FastQC (v0.11.9) [6] to ensure data reliability. Subsequently, TrimGalore (v0.6.6) was employed to remove sequencing adapters and low-quality bases, resulting in high-quality (HQ) reads for further analyses. Cleaned reads were aligned to the human reference genome GRCh38 using the Burrows-Wheeler Aligner (BWA-MEM, v0.7.17). Duplicate reads were marked using Picard Tools (v2.23.8).

### Variant calling and annotation

Variant discovery was conducted using the Genome Analysis Toolkit (GATK) v4.3 [7], implementing best practice pipelines. Base quality score recalibration (BQSR) utilized known indel and SNP sites from the dbSNP and Mills and 1000G gold standard sets. Variant calling was performed per sample using GATK HaplotypeCaller (v4.1.9.0) in gVCF mode, followed by joint genotyping using GATK GenotypeGVCFs. Initial variant filtering employed GATK Variant Quality Score Recalibration (VQSR), with tranche sensitivity set to 99.5% for SNPs and 99.0% for indels. Hard filtering was applied to variants failing VQSR, utilizing thresholds such as QD < 2.0, FS > 60.0, and MQ < 40.0 (for SNPs). The final call set demonstrated a Transition-to-Transversion (Ti/Tv) ratio of 1.99 and an average call rate of 99%. Pedigree checks ensured no unexpected relatedness, and sex checks confirmed reported gender.

The identified variants underwent comprehensive annotation using various databases and tools for biological and clinical interpretation. The RefSeq database facilitated gene identification and variant characterization. Variant annotation was conducted using the Ensembl Variant Effect Predictor (VEP), providing detailed information regarding the functional and biological consequences [8]. Default VEP annotations were complemented with additional plugins, including dbNSFP [9], CADD [10], and Phenotypes [11], to enhance annotation quality. Potential disease associations of variants were investigated using publicly available databases such as OMIM [12], ClinVar [13], and UniProtVar [14]. Population allele frequency data were sourced from the 1000 Genomes Project [15] and gnomAD [16] (encompassing both exome and genome datasets) to effectively distinguish rare variants from common polymorphisms. Functional predictions for mutations were generated using integrated tools within dbNSFP, including SIFT [17], PolyPhen [18], FATHMM [19], MutationTaster [20], MutationAssessor [21], and PROVEAN [22]. Missense variants were further annotated using CADD scores. The SIFT-indel tool [23] evaluated the functional impact of InDels. Furthermore, only canonical transcript-dependent consequences were retained in the final VEP-annotated file to ensure consistency and relevance in downstream analyses.

### Filtering and classification of variants

To identify clinically relevant variants associated with cardiovascular disease, genes linked to “Cardiac Arrhythmias,” “Familial Hypercholesterolemia,” and “Pulmonary Arterial Hypertension” were prioritized using the Genomic England Panel App [24]. These gene panels, typically employed for FH, arrhythmias, and PAH, were utilized to identify potential genetic overlap or secondary risk factors in patients primarily presenting with CAD. Variants within these genes were selected for further investigation.

A multi-step filtering process was implemented to ensure the clinical relevance of the identified variants. Variants exhibiting a population frequency of 1% or less in databases such as gnomAD and the 1000 Genomes Project were retained. The analysis was confined to protein-coding regions and canonical splice sites, prioritizing variants with “HIGH” or “MODERATE” VEP impact. Given the exploratory nature of this study, variants were further filtered using a stringent popmax Allele Frequency (popmax AF) threshold of < 0.1% across all gnomAD subpopulations to enrich for ultra-rare variants while accommodating potential under-representation of the Middle Eastern population.

A comprehensive annotation strategy was employed to assess the potential clinical implications of the filtered variants. Variants were annotated using clinical databases, including ClinVar and UniProtVar, to leverage existing clinical knowledge. Additionally, a combination of standalone and ensemble *in silico* prediction tools, including SIFT, PolyPhen-2, FATHMM, MutationTaster, MutationAssessor, CADD, PROVEAN, and SIFT-Indels, was utilized to predict the impact of nonsynonymous variants, indels, and frameshift variants.

Based on the combined information derived from clinical databases and *in silico* predictions, variants were classified into five categories: Benign, Likely Benign, Variant of Unknown Significance (VUS), Likely Pathogenic, and Pathogenic. This classification process prioritized clinical data over *in silico* predictions. In cases of conflicting clinical annotations, *in silico* predictions were consulted to establish a final classification. For variants absent in clinical databases, stringent criteria based on CADD scores (≥20 for deleterious) and *in silico* tool predictions (consensus from at least three tools) were applied to classify them as predicted deleterious. Variants not meeting these criteria were classified as VUS.

### Functional annotation of candidate variant genes

To elucidate the functional impact of the identified VUS, pathogenic, and likely pathogenic variants, the g:Profiler webserver [25] was employed for enrichment analysis. This tool facilitates the systematic exploration of gene sets by identifying overrepresented GO terms. By focusing on Gene Ontology - Biological Process (GO:BP) terms, Reactome Pathways, and Human Phenotype Ontology, the objective was to uncover the specific molecular and cellular processes influenced by these variants and their potential contributions to cardiovascular disease. The g:SCS (Gene set size corrected) method was applied for multiple testing correction. It is important to note that the results are presented as descriptive functional annotation due to the pre-selected nature of the gene panels, rather than as evidence of statistical enrichment.

### Ethical approval and consent to participate

The study received approval from the institutional ethics committee at the University of Tabuk (Number # UT-91-23-2020). All participants provided written informed consent prior to their involvement in the study.

## Results

### CAD cohort characteristics

This study enrolled 28 patients diagnosed with CAD, providing a detailed profile of this specific cohort. The median age of participants was 53 years, with an interquartile range (IQR) of 40–63 years, indicating a broad age distribution. The study population exhibited a strong male predominance, with 86% of participants being male. Most participants resided in urban areas (75%) and were unemployed (54%). Educational data was available for 16 participants, revealing that 25% had received formal education, while 32% were uneducated. Analysis of cardiovascular risk factors indicated that 57% of participants were non-smokers, while 43% were current smokers. A significant proportion of patients presented with established cardiovascular risk factors, including hypertension (54%) and hyperlipidemia (46%). Moreover, over half of the participants were classified as overweight or obese (54%), and a similar proportion had a diagnosis of diabetes (54%). Regarding angina presentation, the majority (71%) experienced stable angina, while 29% presented with unstable angina. A history of myocardial infarction (MI) was reported in 36% of participants, with 14% experiencing ST-elevation myocardial infarction (STEMI), 14% non-ST-elevation myocardial infarction (NSTEMI), and 3.6% having undergone coronary artery bypass grafting (CABG). A familial history of cardiovascular disease was noted in 36% of the study population.

Laboratory analysis provided a comprehensive metabolic and hematologic profile of patients with coronary artery disease. The cohort exhibited a pronounced dyslipidemic pattern characteristic of elevated cardiovascular risk. Cholesterol levels were suboptimal in a significant proportion of patients. Nearly half (43%, *n* ═ 12) presented with high total cholesterol, with a median level of 215 mg/dl. Only 54% (*n* ═ 15) maintained levels within the optimal range, while one patient (3.6%) was borderline. This pattern was particularly pronounced for low-density lipoprotein cholesterol (LDL-C), the primary atherogenic lipid fraction. The majority of patients (89%, *n* ═ 25) had elevated LDL-C levels, with a median of 142 mg/dl. Optimal LDL-C was observed in only two patients (7.1%). High-density lipoprotein cholesterol (HDL-C), the protective lipid fraction, showed a median level of 45 mg/dl. While half of the cohort (50%, *n* ═ 14) had optimal HDL-C levels, a concerning 29% (*n* ═ 8) displayed levels below the desired range. Triglyceride levels were markedly elevated, with a median of 217 mg/dl. The cohort was evenly divided, with exactly 50% of patients exhibiting high triglyceride levels. C-reactive protein was positive in 32% (*n* ═ 9), with a median level of 2.23 mg/dl. The median hemoglobin level was 14.24 g/dL. A comprehensive summary of the patient characteristics of the study cohort is presented in Table 1.

**Table 1:** Clinical characteristics of the study cohort (*n* ═ 28)

**Biomarker**	***n* (%)**	Median [IQR]*
**Cholesterol**		215.46 [150--280] (mg/dl)
Borderline	1 (3.6%)	
High	12 (43%)	
Optimal	15 (54%)	
**LDL-C**		142.32 [110--205] (mg/dl)
Borderline	1 (3.6%)	
High	25 (89%)	
Optimal	2 (7.1%)	
**HDL-C**		45.67 [35--62] (mg/dl)
Abnormal	8 (29%)	
Optimal	14 (50%)	
Unknown	6 (21%)	
**Triglycerides**		217.21 [120--457] (mg/dl)
High	14 (50%)	
Optimal	14 (50%)	
**C-reactive protein**		2.23 [0.6--4.5] (mg/dl)
Unknown	7 (25%)	
Negative	12 (43%)	
Positive	9 (32%)	
**Hemoglobin**		14.24 [9.5--17.9] (g/dl)
Low	1 (3.57%)	
Optimal	27 (96.42%)	

*IQR: Interquartile Range of the measured parameter in the cohort.

### Identified variants in the cohort

Whole-exome sequencing analysis of the study cohort identified variants within 74 genes that overlapped with the pre-defined GenePanel. This analysis generated a total of 8,251 variants across the cohort. When categorized by the associated condition, 1,020 variants were related to the Familial Hypercholesterolemia gene panel, 5,632 to the Cardiac Arrhythmias gene panel, and 1,599 to the Pulmonary Arterial Hypertension gene panel.

**Figure 1.**
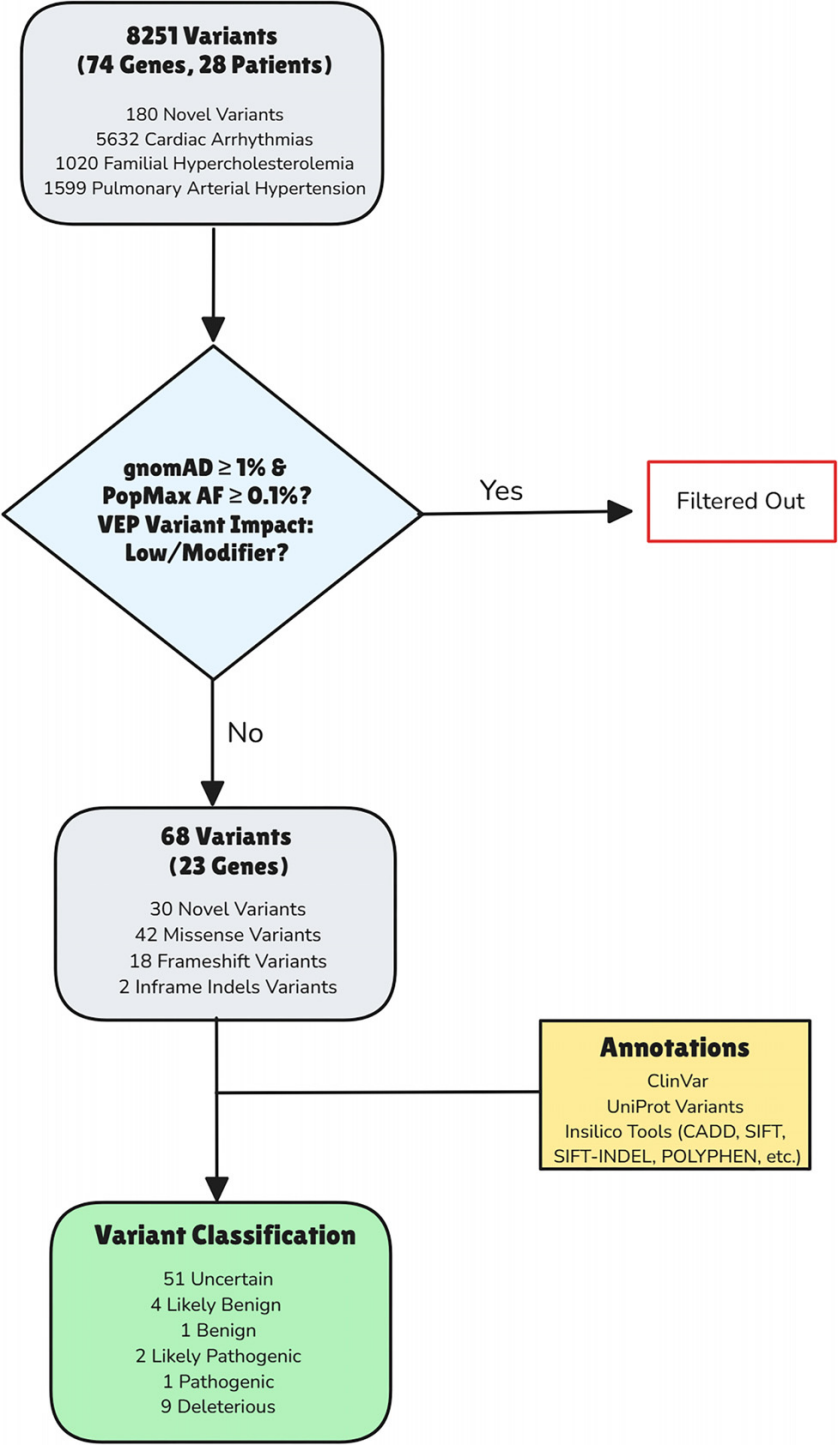
Workflow illustrating the steps involved in the identification, filtering, and classification of variants associated with cardiac arrhythmias, familial hypercholesterolemia, and pulmonary arterial hypertension through whole-exome sequencing.

**Figure 2.**
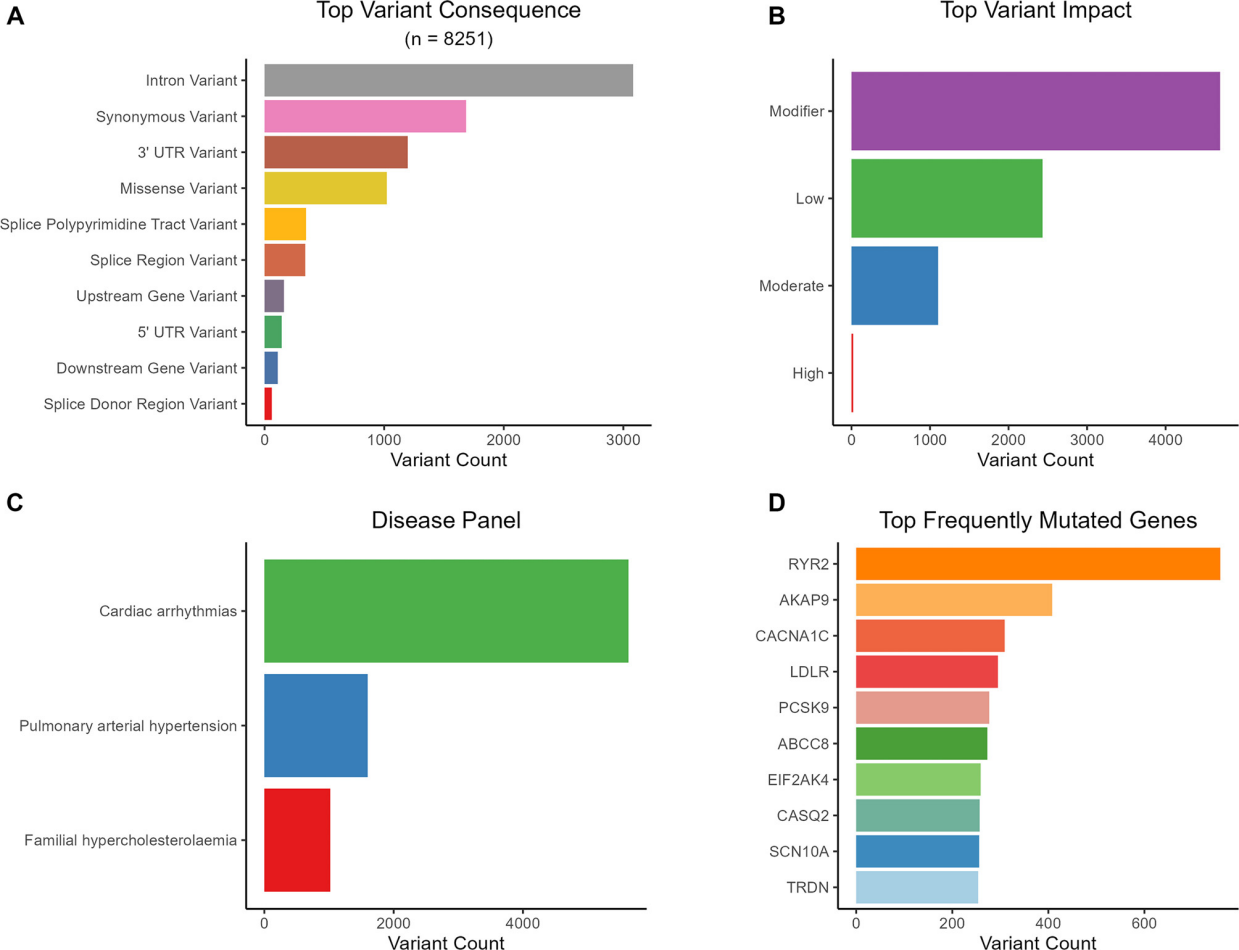
**Summary of panel-overlapping variants identified by WES in the obstructive CAD cohort.** (A) Top VEP consequence categories across 8,251 variants within 74 PanelApp-curated genes. (B) VEP impact distribution (modifier, low, moderate, high). (C) Variant counts by disease panel (CA, PAH, FH). (D) Top frequently mutated genes in the unfiltered dataset (including RYR2, AKAP9, CACNA1C, LDLR, and PCSK9). Abbreviations: CA: Cardiac arrhythmias; CAD: Coronary artery disease; FH: Familial hypercholesterolaemia; PAH: Pulmonary arterial hypertension; PanelApp: Genomics England PanelApp; VEP: Variant Effect Predictor; WES: Whole-exome sequencing.

Analysis of variant distribution by genomic location revealed that the majority of these variants were either intronic (37%) or synonymous (20%). Among the remaining variants, 991 were missense variants (12%), 1,197 were located in the 3′ UTR, 143 in the 5′ UTR, and 18 were frameshift variants. Notably, 180 variants (2%) were classified as novel, having not been previously reported in public databases such as dbSNP or COSMIC. The Variant Effect Predictor (VEP) impact categorization assigned 21 variants as high impact, 2,433 as low impact, 1,103 as moderate impact, and 4,694 as modifier impact. In this initial, unfiltered variant dataset, the most frequently mutated genes were *RYR2*, followed by *AKAP9*, *CACNA1C*, *LDLR*, and *PCSK9*. The methodology employed in the study to identify candidate variants is illustrated in Figure 1. A key summary of the identified variants within the study cohort is provided in Figure 2.

### Candidate variant detection and classification

**Figure 3.**
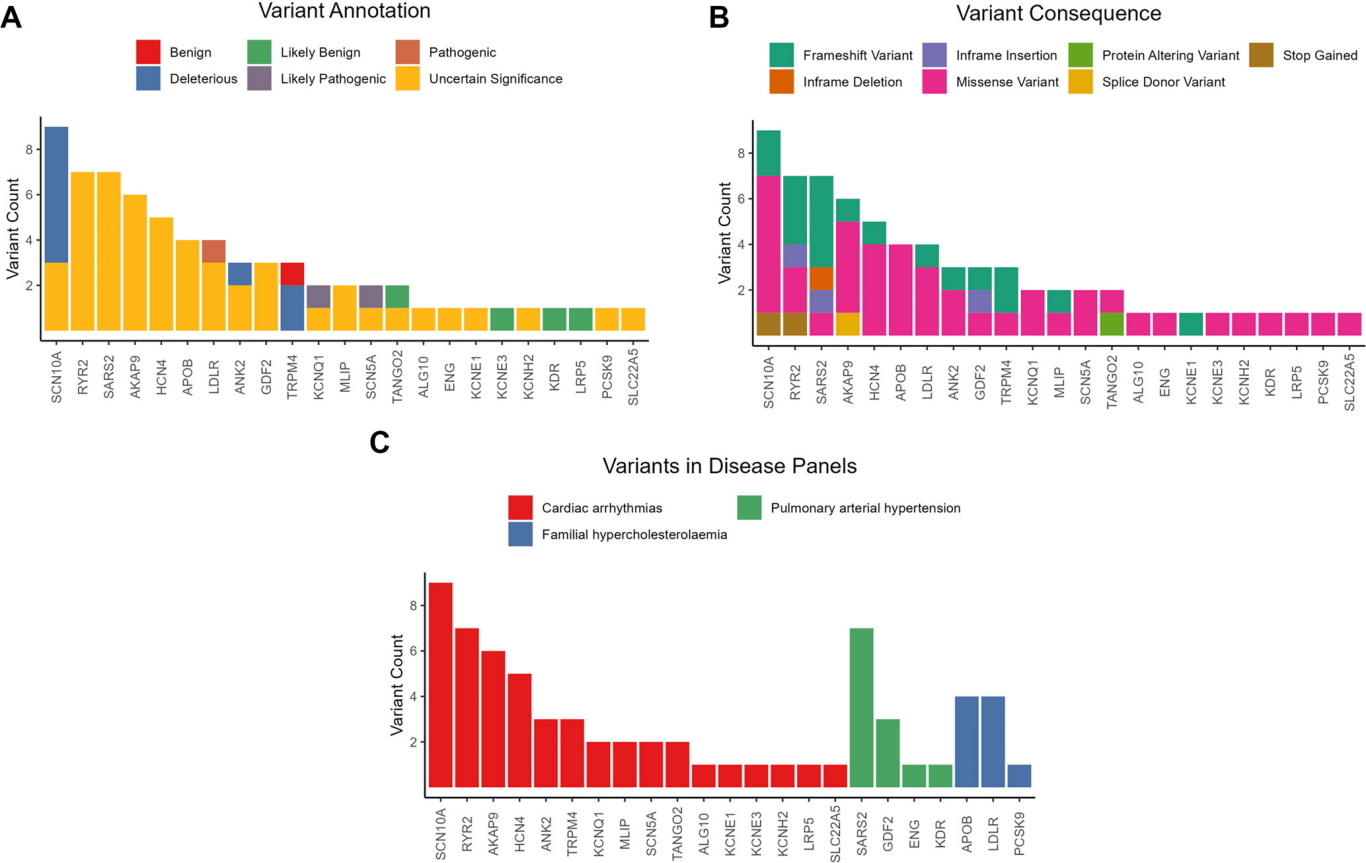
**Candidate variant classification and distribution after rare-variant filtering.** Following PopMax AF <0.1% and VEP impact filtering of PanelApp FH/CA/PAH genes in WES data from a CAD cohort (*n* ═ 28), 68 candidate variants across 23 genes were retained (30/68 novel). (A) Stacked per-gene counts by final classification (B, LB, VUS, D, LP, P; overall: VUS 51/68, D 9/68, LB 4/68, B 1/68, LP 2/68, P 1/68) based on ClinVar/UniProtVar evidence and in silico predictions. (B) Stacked per-gene counts by consequence category, dominated by missense (42/68) and frameshift (18/68) variants. (C) Candidate variants mapped to PanelApp panels (CA 47/68; PAH 12/68; FH 9/68); genes are ordered by total candidate count (SCN10A highest). Abbreviations: B: Benign; CA: Cardiac arrhythmias; CAD: Coronary artery disease; ClinVar: Clinical Variation database; D: Predicted deleterious; FH: Familial hypercholesterolaemia; LB: Likely benign; LP: Likely pathogenic; P: Pathogenic; PAH: Pulmonary arterial hypertension; PanelApp: Genomics England PanelApp; PopMax AF: Population maximum allele frequency; UniProtVar: UniProt variant annotations; VEP: Variant effect predictor; VUS: Variant of uncertain significance; WES: Whole-exome sequencing.

After applying initial filters based on population frequency and VEP impact, we refined the dataset further. By using a strict PopMax Allele Frequency (AF) threshold, we significantly narrowed the investigation’s focus. This approach effectively removed common variations and noise, resulting in 68 potential rare variants spread across 23 different genes for further analysis. A notable proportion of these candidates, 30 variants (44%), were novel, indicating they had not been previously documented in public databases. The predominant variant type was missense (*n* ═ 42, 62%), followed by frameshift mutations (*n* ═ 18, 26%). The remaining variants included in-frame indels, stop gain mutations, splice site variants, and other protein-altering variations. To evaluate the potential clinical relevance of these candidate variants, annotations were generated using ClinVar, UniProtVar, and a range of *in silico* prediction tools. This assessment resulted in the classification of the variants into distinct categories. Variants of unknown significance (VUS) constituted the largest group (*n* ═ 51; 75%), emphasizing the need for further investigation to elucidate their clinical implications. Other classifications included likely benign (LB, *n* ═ 4; 6%), benign (B, *n* ═ 1; 1.5%), predicted deleterious (D, *n* ═ 9; 13%), likely pathogenic (LP, *n* ═ 2; 3%), and a single pathogenic variant (P, *n* ═ 1; 1.5%). The majority of classified variants were associated with the Cardiac Arrhythmias gene panel (*n* ═ 47; 69%), Pulmonary Arterial Hypertension (*n* ═ 12; 18%), and Familial Hypercholesterolemia (*n* ═ 9; 13%). The *SCN10A*gene (*n* ═ 9) harbored the highest number of candidate variants, followed by *RYR2*, *SARS2*, *AKAP9*, and *HCN4*. A comprehensive overview of the candidate variants identified in each sample is presented in Figure 3.

### Rare and potentially pathogenic variant identification

A detailed analysis of the candidate variants identified 12 variants classified as either pathogenic (*n* ═ 1), likely pathogenic (*n* ═ 2), or predicted deleterious (*n* ═ 9), suggesting a potential role in the development of cardiovascular disease. These variants were observed in 8 individual patients, with each patient carrying at least one such variant. Notably, 11 of these variants were unique to individual patients, while one variant, *TRPM4* (ENST00000252826.10:c.247dup), was recurrent, identified in two separate patients. In the context of Familial Hypercholesterolemia, the *LDLR* variant (rs879254847; ENST00000558518.6:c.1255T>G) was classified as pathogenic in one patient. A comprehensive summary of the unique pathogenic, likely pathogenic, and predicted deleterious variants identified within this cohort is provided in Table 2.

**Table 2:** Key pathogenic, likely pathogenic, and predicted deleterious variants identified in genes associated with the genomic england genepanelapp for cardiac arrhythmias, familial hypercholesterolemia, and pulmonary arterial hypertension

**Gene**	**GeneApp Panel** *^2^*	**Variant ID**	**Variant**	**Variant type**	**ClinVar** *^1^*	**UniProtVar** *^1^*	**In silico prediction** *^1^*	**Interpreted classification** *^1^*
ANK2	CA	COSV100003794	ENST00000357077.9:c.2693G>T:p.Ser898Ile	Missense variant	-	-	D	D
KCNQ1	CA	rs199472737	ENST00000155840.12:c.877C>T:p.Arg293Cys	Missense variant	US	LP/P	D	LP
LDLR	FH	rs879254847	ENST00000558518.6:c.1255T>G:p.Tyr419Asp	Missense variant	P/LP	-	D	P
SCN10A	CA	Novel	ENST00000449082.3:c.1200T>A:p.Tyr400Ter	Stop gained	-	-	D	D
SCN10A	CA	Novel	ENST00000449082.3:c.4473C>G:p.Ile1491Met	Missense variant	-	-	D	D
SCN10A	CA	Novel	ENST00000449082.3:c.4467C>G:p.Asn1489Lys	Missense variant	-	-	D	D
SCN10A	CA	Novel	ENST00000449082.3:c.4458C>G:p.Ile1486Met	Missense variant	-	-	D	D
SCN10A	CA	Novel	ENST00000449082.3:c.4453C>A:p.Leu1485Ile	Missense variant	-	-	D	D
SCN10A	CA	rs770288343	ENST00000449082.3:c.5339C>T:p.Pro1780Leu	Missense variant	-	-	D	D
SCN5A	CA	rs199473124	ENST00000423572.7:c.1700T>A:p.Leu567Gln	Missense variant	US	LP/P	N	LP
TRPM4	CA	rs754625848	ENST00000252826.10:c.247dup:p.Ala83GlyfsTer13	Frameshift variant	-	-	D	D

Abbreviations: ^1^D: Predicted deleterious; N: Neutral; P: Pathogenic; LP: Likely pathogenic; VUS: Variant of unknown significance; ^2^CA: Cardiac arrhythmias; FH: Familial hypercholesterolaemia.

### Functional characterization of candidate variants

To further investigate the functional implications of the identified pathogenic, likely pathogenic, and VUS variants, a comprehensive functional annotation analysis was conducted using GO, Reactome Pathways, and HPO. The GO:BP analysis highlighted a concentration of genes involved in cardiac muscle cell action potential (*P* ═ 1.2 × 10^--16^) and metal ion transport (*P* ═ 4.2 × 10^--9^), driven by key regulators such as KCNQ1, SCN5A, and KCNH2. Reactome pathway analysis further supported these findings, demonstrating enrichment in pathways related to Cardiac Conduction (*P* ═ 1.6 × 10^--7^), Muscle Contraction (*P* ═ 3.6 × 10^--6^), Phase 3 - Rapid Repolarization (*P* ═ 1.6 × 10^--7^), and LDL Clearance (*P* ═ 9.7 × 10^--4^).

**Figure 4.**
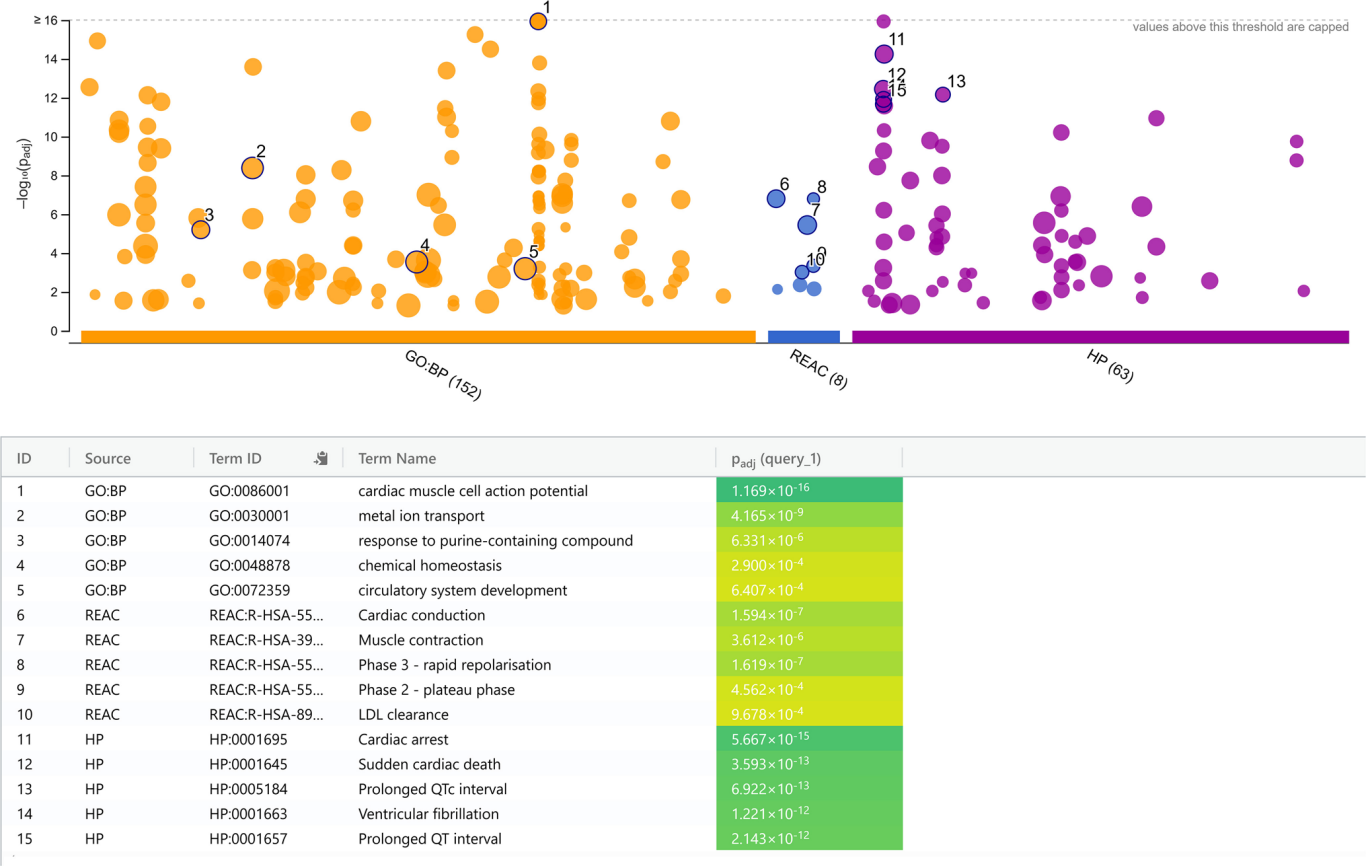
**GO/REAC/HP functional annotation of candidate genes.** g:Profiler Manhattan plot showing GO:BP terms (orange), REAC pathways (blue) and HP terms (purple) for the candidate-gene set, plotted as --log_10_(padj) (g:SCS). Values >16 are capped. The numbered points correspond to the top terms listed in the table below, including GO:BP signals for cardiac muscle cell action potential and metal ion transport; REAC signals for cardiac conduction, muscle contraction, phase 3—rapid repolarisation and LDL clearance; and HP signals for cardiac arrest, sudden cardiac death, prolonged QTc interval and ventricular fibrillation. Abbreviations: GO: Gene Ontology; GO:BP: Gene Ontology—Biological Process; g:SCS: Gene set size–corrected significance threshold; HP: Human Phenotype Ontology (g:Profiler source label); HPO: Human Phenotype Ontology; LDL: Low-density lipoprotein; p_adj_: Adjusted *P* value; REAC: Reactome pathways.

The functional annotation by HPO provided insights into the potential clinical manifestations associated with these variants. The genes were significantly enriched for phenotypes related to severe cardiac dysfunction, including Cardiac Arrest (*P* ═ 5.7 × 10^--15^), Sudden Cardiac Death (*P* ═ 3.6 × 10^--13^), Prolonged QTc Interval (*P* ═ 6.9 × 10^--13^), and Ventricular Fibrillation (*P* ═ 1.2 × 10^--12^). Functional annotation of the candidate genes confirmed that the identified variants fall into primary functional categories related to cardiac muscle function, ion transport, and lipid metabolism, consistent with the initial selection criteria of the gene panels. Figure 4 provides a visual representation of the aggregated GO terms, Reactome pathways, and HPO terms, illustrating the functional impact of the identified variants. Collectively, these annotations, summarized in Table 3, confirm the high functional relevance of the identified variants in maintaining cardiac rhythm and their potential contribution to severe arrhythmic phenotypes.

## Discussion

CAD is the third leading cause of death worldwide, responsible for an estimated 17.8 million fatalities annually. Individuals with existing CAD often experience a diminished quality of life. In 2022, global statistics indicated approximately 315 million cases of CAD [26, 27]. The etiology of CAD is multifactorial, involving a complex interplay of modifiable and non-modifiable risk factors. Atherosclerosis typically begins in younger populations and is influenced by various factors, with abnormal lipid metabolism being a primary contributor. As individuals age, the severity and prevalence of CAD increase, particularly among those over 75 years old, who face a heightened risk of multi-vessel CAD [28]. The median age of our cohort was 53 years, indicating a significant CAD burden on a relatively younger population. Nevertheless, optimal management of modifiable risk factors may mitigate the impact of non-modifiable risk factors associated with CAD. A preventive program targeting individuals aged 70 and older, identified as high-risk for CVD, demonstrated a substantial risk reduction of 13%–20% attributable to improved management of hypertension and hypercholesterolemia [29].

Sex-based biological variations arise from a complex interaction among sex chromosomes, hormones, and environmental influences, leading to different molecular mechanisms that affect the phenotypic manifestation of CAD and atherosclerosis. Estrogens and androgens (specifically estradiol and testosterone) interact with their respective receptors present in all cardiovascular tissues [30]. Estrogen is believed to offer protective effects against atherosclerosis, contributing to the lower incidence of cardiovascular disease in premenopausal women, with risk increasing post-menopause [31]. Our study reveals a higher incidence of CAD in males compared to females, indicating a natural gender predisposition to the disease. Traditionally, CAD was perceived as predominantly affecting men, as morbidity and mortality rates are generally higher in this group, while women experience lower incidence rates [32]. However, since women are often diagnosed with CAD approximately 10 years later than men, this delay may result in greater comorbid conditions associated with disease severity at diagnosis [33]. Additionally, research indicates that atherosclerotic plaques in women are typically fibrous and stable, while those in men tend to be more atheromatous, featuring higher levels of inflammatory cells, calcification, lipids, and hemorrhage [34].

Our findings reveal a consistent pattern of pathological risk factors, including hypertension, hyperlipidemia, obesity, T2DM and familial history, prevalent in a substantial proportion of the CAD cohort. Elevated blood pressure stands out as a significant modifiable risk factor for all forms of CAD, contributing to 90% of the attributable risk for myocardial infarction in men and 94% in women [35]. The intricate pathophysiological mechanisms linking blood pressure to CAD encompass its role as a physical force affecting atherosclerotic plaque formation, as well as interactions among pulsatile hemodynamics, arterial stiffness, and coronary perfusion [36]. Effective treatment of arterial hypertension has been shown to reduce the risk of coronary events in individuals without CAD [37]. The benefits of lowering blood pressure in patients with existing CAD are believed to surpass those associated with specific medications. Research suggests that maintaining systolic blood pressure at 115 mmHg and diastolic blood pressure at 75 mmHg is associated with a reduced risk of mortality from CAD. Furthermore, a reduction of systolic blood pressure by 20 mmHg can decrease mortality risk by 33%–50% in both men and women aged 40–89 [38]. Elevated levels of serum lipids, such as LDL cholesterol and triglycerides, are known to heighten the risk of heart disease due to plaque accumulation in arteries [39]. While lifestyle factors often contribute to hyperlipidemia, genetic variations also play a significant role [40]. Approximately half of individuals with early-onset CAD exhibit dyslipidemia and a family history, primarily characterized by elevated LDL cholesterol and/or triglycerides [41]. Notably, research has demonstrated that lipidomic profiles in familial hyperlipidemias closely resemble those found in population-based hyperlipidemias, suggesting significant overlap in underlying mechanisms [42]. The risk of CAD appears remarkably similar regardless of whether hyperlipidemic individuals originate from families with high prevalence of the condition or from the general population.

**Table 3:** Descriptive functional enrichment of candidate genes across GO, reactome, and HPO databases

Term category*	**Enriched term**	**Term ID**	**p_adj_** **(g:SCS)**	**Enriched genes**
GO:BP	cardiac muscle cell action potential	GO:0086001	1.2 × 10^--16^	KCNQ1,SCN10A,HCN4,SCN5A,KCNE1,RYR2,ANK2,TRPM4,KCNH2,AKAP9
GO:BP	metal ion transport	GO:0030001	4.2 × 10^--9^	KCNQ1,SCN10A,HCN4,SCN5A,KCNE1,PCSK9,RYR2,ANK2,SLC22A5,TRPM4,KCNH2, AKAP9
GO:BP	response to purine-containing compound	GO:0014074	6.3 × 10^--6^	KCNQ1,HCN4, KCNE1,RYR2, TRPM4,AKAP9
GO:BP	chemical homeostasis	GO:0048878	2.9 × 10^--4^	KCNQ1,GDF2, LDLR,APOB,PCSK9,RYR2,ANK2,TRPM4,KCNH2
GO:BP	circulatory system development	GO:0072359	6.4 × 10^--4^	KCNQ1,GDF2, HCN4,LDLR,APOB,SCN5A,RYR2,ANK2,ENG
REAC	Cardiac conduction	REAC:R-HSA- 5576891	1.6 × 10^--7^	KCNQ1,SCN10A,SCN5A,KCNE1,RYR2,KCNH2,AKAP9
REAC	Phase 3 - rapid repolarization	REAC:R-HSA- 5576890	1.6 × 10^--7^	KCNQ1,KCNE1,KCNH2,AKAP9
REAC	Muscle contraction	REAC:R-HSA- 397014	3.6 × 10^--6^	KCNQ1,SCN10A,SCN5A,KCNE1,RYR2,KCNH2,AKAP9
REAC	Phase 2 - plateau phase	REAC:R-HSA- 5576893	4.6 × 10^--4^	KCNQ1,KCNE1,AKAP9
REAC	LDL clearance	REAC:R-HSA- 8964038	9.7 × 10^--4^	LDLR,APOB,PCSK9
HP	Cardiac arrest	HP:0001695	5.7 × 10^--15^	KCNQ1,SCN10A,HCN4,LDLR,APOB,SCN5A,KCNE1,PCSK9,RYR2,ANK2,TRPM4,KCNH2,AKAP9,TANGO2
HP	Sudden cardiac death	HP:0001645	3.6 × 10^--13^	KCNQ1,SCN10A,HCN4,LDLR,APOB,SCN5A,KCNE1,PCSK9,RYR2,ANK2,KCNH2,AKAP9
HP	Prolonged QTc interval	HP:0005184	6.9 × 10^--13^	KCNQ1,SCN10A,HCN4,SCN5A,KCNE1,ANK2,KCNH2, AKAP9,TANGO2
HP	Ventricular fibrillation	HP:0001663	1.2 × 10^--12^	KCNQ1,SCN10A,HCN4,SCN5A,KCNE1,RYR2,TRPM4, KCNH2,AKAP9,TANGO2
HP	Prolonged QT interval	HP:0001657	2.1 × 10^--12^	KCNQ1,SCN10A,HCN4,SCN5A,KCNE1,ANK2,TRPM4, KCNH2,AKAP9,TANGO2

Results are ranked using the g:SCS multiple-te sting correction. Abbreviations: ^*^GO: Gene ontology; BP: Biological process; REAC: Reactome; HP: Human phenotype.

Obesity is independently associated with the development of coronary heart disease (CHD), and weight reduction has been shown to improve risk factors and prognosis [43, 44]. Interestingly, literature indicates that metabolically healthy obesity, characterized by the absence of cardiometabolic risk factors such as insulin resistance, dyslipidemia, hypertension, and type 2 diabetes, does not appear to increase the risk of atherosclerosis. However, some studies suggest that individuals who are obese but metabolically healthy may later develop metabolically unhealthy obesity [45, 46]. In addition to being a significant independent risk factor, type 2 diabetes is commonly associated with pathologies such as hypertension and dyslipidemia, which are known risk factors for atherosclerotic cardiovascular disease [47]. Hyperglycemia contributes to cardiovascular disease development through mechanisms like advanced glycation end product production and elevated oxidative stress [48]. The progression of CAD to heart failure is significantly influenced by insulin resistance and hyperglycemia [49]. Evidence suggests that managing individual cardiovascular risk factors can help prevent or reduce the onset of CAD in diabetic individuals [50]. Furthermore, significant benefits are observed when multiple cardiovascular risk factors—glycemic control, blood pressure management, and lipid regulation—are concurrently addressed [51]. A family history of CAD is an increasingly recognized cardiovascular risk factor that extends beyond its traditional role as a modifier of disease risk [52]. First-degree relatives of individuals with CAD, particularly siblings, face a significantly higher risk of developing the disease at a younger age [53]. Recent guidelines from the Canadian Cardiovascular Society (CCS) and the European Society of Cardiology (ESC) now incorporate family history of CAD as an essential criterion for calculating risk factor-weighted clinical likelihood (RF-CL) during pre-test assessments, underscoring the relevance of this medical history [54].

Genetic variability is acknowledged to affect cellular mechanisms, influencing susceptibility to various complex diseases, including CAD [55]. Whole-exome sequencing, a technique utilized for genetic correlation studies, can aid in identifying molecular abnormalities associated with CAD [56]. In our cohort, next-generation whole exome sequencing identified a total of 8,251 variants across 74 genes. These variants were categorized into panels associated with familial hypercholesterolemia, cardiac arrhythmias, and pulmonary arterial hypertension. Among these, 180 variants (2%) were classified as novel, and 21 variants were deemed high-impact according to VEP. After filtering, variants in genes associated with cardiac arrhythmias (*n* ═ 47; 69%) represented the majority of identified candidates, followed by those linked to pulmonary arterial hypertension (*n* ═ 12; 18%) and familial hypercholesterolemia (*n* ═ 9; 13%). While our cohort comprised patients with primary CAD, we identified a notable burden of variants in genes traditionally associated with cardiac arrhythmias (e.g., SCN10A, ANK2) and pulmonary hypertension. It is crucial to understand that the presence of these variants does not necessarily indicate an active clinical diagnosis of arrhythmia or PAH in these patients; instead, these findings may signify latent genetic susceptibility. In the context of ischemic heart disease, such variants could act as distinct risk modifiers, potentially lowering the threshold for arrhythmias under ischemic stress or impacting long-term prognosis. Carrying a hidden ‘pro-arrhythmic’ genetic burden, such as mutations in *SCN5A* or *KCNQ1*, may heighten the risk of ventricular fibrillation during ischemic events, which could account for the increased incidence of sudden cardiac death observed in certain CAD patient populations. Similarly, PAH-associated variants may predispose individuals to adverse vascular changes, influencing the coronary vasculature’s response to prolonged ischemia. This underscores the importance of multi-panel whole exome sequencing in uncovering ‘silent’ genetic risks that may be overlooked in single-phenotype screening.

Evidence of molecular etiology has demonstrated that microstructural abnormalities (e.g., partial or complete lack of structures, fatty and/or fibrous replacement of normal tissues, calcification) or functional abnormalities of the action potential may be primary causes of arrhythmogenic diseases [57]. Diagnostic tachycardias or bradycardias may stem from a genetic predisposition to these pathophysiological alterations. Reports of familial clustering of prevalent cardiac arrhythmias have confirmed the genetic basis of these conditions [58]. Serum LDL cholesterol levels that are elevated, alongside an increased risk of coronary artery disease, are associated with familial hypercholesterolemia, a Mendelian disorder [59]. This disease arises from pathogenic DNA variations in any of three associated genes: LDLR, APOB, or PCSK9. Large-scale population studies utilizing gene sequencing have revealed that mutations linked to familial hypercholesterolemia are present in 0.2% to 0.5% of the general population and up to 2% of individuals with early-onset CAD [60]. A pathogenic variant in the *LDLR* gene has been identified in this study as being associated with familial hypercholesterolemia. Chronic hypertension can lead to myocardial infarction, atrial fibrillation, congestive heart failure, and left ventricular hypertrophy, among other detrimental alterations in the anatomy and physiology of the heart [61]. The identification of several Mendelian types of hypertension has been crucial for understanding the mechanisms that regulate blood pressure and increase the risk of coronary artery disease [62].

Our analysis identified the APOB gene as harboring the highest number of candidate variants, followed by SCN10A, AKAP9, ANK2, and RYR2. Apolipoprotein B (APOB) is vital for lipoprotein assembly and secretion, including very low-density lipoprotein (VLDL) and LDL [63]. Elevated levels of APOB are correlated with an increased risk of CAD [64]. Such high APOB levels indicate a greater number of atherogenic particles in circulation, a known contributor to atherosclerosis, consistent with the clinical presentation of our cohort. Numerous variants in the APOB gene have been investigated for their associations with CAD and related risk factors. These polymorphisms can influence APOB levels, lipid profiles, and CAD risk. For example, the APOB gene polymorphism c.12669G>A, which results in the amino acid substitution p.Gln4154Lys, has been studied across various populations [65]. A study involving the Indian population revealed that the frequency of the R-(mutant) allele was significantly higher in CAD patients compared to healthy controls [65]. Similarly, a study of Mexican patients with CAD found that the frequency of the X+/X+ genotype in the Xba I polymorphism of the APOB gene was significantly elevated in CAD patients relative to controls [66]. Additionally, the R3500Q mutation in the APOB gene is responsible for familial defective apolipoprotein B-100, which is associated with heightened LDL cholesterol levels and coronary artery calcification. Research in the Old Order Amish population indicated a high carrier frequency of the R3500Q mutation (12%); consequently, carriers exhibited significantly higher LDL cholesterol levels and a greater likelihood of extensive coronary artery calcification [67]. The SCN10A gene encodes the Nav1.8 protein, a voltage-gated sodium channel that regulates nerve and muscle cell excitability [68]. This protein belongs to the voltage-gated sodium channel family, responsible for the rapid influx of sodium ions into cells during the depolarization phase of the action potential. Certain common and rare SCN10A variants have been linked to Brugada Syndrome, a hereditary channelopathy resulting from genetically programmed loss-of-function in the cardiac sodium channel [69]. The allele rs6795970 (V1073) has been shown to increase the incidence of this syndrome by inducing electrophysiological abnormalities, such as a positive shift in steady-state activation and slower recovery following inactivation [70]. Notably, our study identified five novel variants in SCN10A classified as predicted deleterious. Given SCN10A’s functional relationship to cardiac arrhythmias, these novel variants warrant further investigation to clarify their roles in modulating cardiovascular risk or susceptibility to arrhythmic events in CAD patients. A-kinase anchoring protein 9 (AKAP9) is a scaffolding protein involved in cellular signaling, particularly with cAMP/PKA pathways, and has been implicated in various diseases, including cardiovascular conditions [71]. These pathways regulate the intensity, duration, and compartmentalization of nucleotide-dependent signaling, thus establishing localized cAMP pools [72]. Several members of the AKAP family are expressed in the cardiovascular system, directing critical processes such as endothelial barrier function and excitation-contraction coupling, which are essential for maintaining homeostatic functioning of the heart and vasculature [72]. AKAP9 has been identified as a candidate gene in arrhythmogenic diseases, with certain genetic variations associated with increased risk for SCN5A-negative Brugada Syndrome [73] and dilated cardiomyopathy [74]. The ANK2 gene encodes ankyrin-B, a protein essential for accurate membrane protein targeting in cardiac tissues [75]. Through its adapter function, ankyrin-B facilitates the proper localization of vital cardiac cell components, including ion channels, transporters, receptors, and signaling molecules, thus maintaining normal heart rhythms and performance [75]. The human ANK2 variants R990Q, E1425G, V1516D, and R1788W result in ankyrin-B loss-of-function, leading to ‘Ankyrin-B syndrome.’ This disorder presents with bradycardia, heart rate variability, conduction block, atrial fibrillation, prolonged QT intervals, and the potential for lethal catecholaminergic polymorphic ventricular tachycardia and sudden cardiac death [76]. A predicted deleterious variant was identified in the ANK2 gene, a well-established susceptibility gene for cardiac arrhythmias. The RYR2 gene encodes the sarcoplasmic reticulum cardiac ryanodine receptor/calcium release channel RyR2, which is essential for maintaining intracellular calcium levels and regulating cardiac excitation–contraction coupling [77]. Research indicates that pathogenic RYR2 mutations predominantly manifest as missense variants (86%–92%), with RyR2 exhibiting limited tolerance for genetic variants that induce loss-of-function defects [78]. Functional characterization of the variants identified in our study revealed various potential impairments that could affect cardiac structural and functional efficiency. These include aberrant cardiac muscle cell action potential, actin-mediated cell contraction, and cardiac conduction, particularly during phase 3 rapid repolarization, which may predispose individuals to clinical manifestations such as ventricular fibrillation, arrhythmia, cardiac arrest, and sudden cardiac death. Furthermore, likely pathogenic variants have been reported in our cohort for the *SCN5A* and *KCNQ1* genes. The SCN5A gene encodes the principal voltage-gated sodium channel responsible for initiating and propagating the cardiac action potential. Pathogenic variants in SCN5A were previously linked exclusively to primary electrical disorders or channelopathies. Contemporary evidence, however, indicates a significant association between SCN5A dysfunction and the development of structural cardiomyopathies [79]. Additionally, characterizing KCNQ1 variants has been shown to enhance risk stratification in patients with cardiac arrhythmias [80].

### Limitations

While our study provides valuable insights into the genetic landscape of this CAD cohort, several limitations must be acknowledged. First, the study cohort comprised only 28 patients, a size sufficient for exploratory descriptive analysis of rare-variant burden but inadequate for establishing formal genotype-phenotype associations or calculating statistical significance regarding disease risk. Furthermore, the absence of a locally sequenced healthy control group constitutes a significant limitation. To address this, we utilized the gnomAD global database as a reference for population allele frequencies. However, we recognize that gnomAD may not fully represent the specific genetic background of Middle Eastern populations, potentially affecting our assessment of variant rarity. Additionally, variants classified as ‘predicted deleterious’ or ‘VUS’ were determined based on *in silico* predictions and existing database evidence. Without *in vitro* functional assays or segregation analysis within families, the precise biological impact of these novel variants on protein function remains speculative. Finally, our analysis was limited to 74 genes across three specific panels (FH, CA, and PAH), which may have resulted in the oversight of other relevant genetic factors contributing to CAD outside these predefined pathways.

## Conclusion

In conclusion, our study employed Whole Exome Sequencing to uncover a significant burden of rare genetic variants within a cohort of 28 patients with Coronary Artery Disease. By applying a stringent population-based filter (PopMax AF < 0.1%), we identified 68 high-confidence candidate variants across 23 genes associated with lipid metabolism, cardiac rhythm, and vascular function. The identification of a pathogenic *LDLR* variant and likely pathogenic variants in *SCN5A* and *KCNQ1* underscores the presence of clinically actionable genetic drivers that may exacerbate CAD progression or increase the risk of secondary complications such as arrhythmias. Furthermore, the discovery of nine predicted deleterious variants, including five novel mutations in the *SCN10A* gene, highlights the potential role of latent genetic modifiers in underrepresented populations. These findings illustrate that genomic screening can reveal underlying predispositions that standard clinical assessments may overlook, advancing a more personalized approach to managing cardiovascular risk.

## Data Availability

The data supporting the reported results can be requested from the corresponding author through the email (rashid@ut.edu.sa).
